# Impacts of Groundwater Recharge from Rubber Dams on the Hydrogeological Environment in Luoyang Basin, China

**DOI:** 10.1155/2014/183457

**Published:** 2014-07-14

**Authors:** Shaogang Dong, Baiwei Liu, Huamin Liu, Shidong Wang, Lixin Wang

**Affiliations:** ^1^College of Environment and Resources, Inner Mongolia University, Hohhot 010021, China; ^2^School of Environmental Studies, China University of Geosciences, Wuhan 430074, China; ^3^College of Life Sciences, Inner Mongolia University, Hohhot 010021, China; ^4^National Secondary Occupation School, Xilingol Vocational College, Xilinhot 026000, China

## Abstract

In the rubber dam's impact area, the groundwater total hardness (TH) has declined since 2000, ultimately dropping to 100–300 mg/L in 2012. pH levels have shown no obvious changes. NH_4_-N concentration in the groundwater remained stable from 2000 to 2006, but it increased from 2007 to 2012, with the largest increase up to 0.2 mg/L. NO_3_-N concentration in the groundwater generally declined in 2000–2006 and then increased from 2007; the largest increase was to 10 mg/L in 2012. Total dissolved solids (TDS) of the groundwater showed a general trend of decline from 2000 to 2009, but levels increased after 2010, especially along the south bank of the Luohe River where the largest increase recorded was approximately 100 mg/L. This study has shown that the increases in the concentrations of NH_4_-N and NO_3_-N were probably caused by changes in groundwater levels. Nitrates adsorbed by the silt clay of aeration zone appear to have entered the groundwater through physical and chemical reactions. TDS increased because of groundwater evaporation and some soluble ions entered the groundwater in the unsaturated zone. The distance of the contaminant to the surface of the aquifer became shorter due to the shallow depth of groundwater, resulting in the observed rise in pollutant concentrations more pronounced.

## 1. Introduction

Groundwater is an important water resource because of its wide distribution, good quality, ease of access, and small seasonal shifts. It becomes critical especially in arid and semiarid areas, as it may often be the only water source. The chemical compositions of the natural groundwater developed during the long geological history. It is affected by the types and characters of rocks to which the water is exposed, the feature of the replenishment water, and the water-rock interactions. As human actions on the environment have intensified, they have become a primary cause of the impact on the chemical characteristics of the groundwater in certain locations. For example, groundwater has been overpumped for industrial use and agriculture, which has caused the groundwater levels to decline and quality to deteriorate [1]. The concentration of nitrogen of the regional groundwater was raised by over fertilization in agriculture [2–4]. Insecticide residue polluted the surface water and shallow groundwater [5, 6]. Solid municipal waste and industrially manufactured solid waste increased the organic, heavy metals, and inorganic groundwater ions [7–9]. Excess discharge of domestic and industrial wastewater has also polluted the groundwater [10]. Groundwater overpumping in coastal areas has caused seawater intrusion [11–13].

Since 1965, more than 1000 rubber dams have been built in China for various purposes, such as irrigation, hydropower, groundwater replenishment, flood control, beautification of the environment, and recreation [14]. There are still a certain number of rubber dams under construction and being planned, especially in the arid and semiarid parts of China. The construction of rubber dams has changed the characteristics of the regional groundwater flow system and increased the amount of groundwater. It has also caused a series of environmental problems. The rise in the groundwater table has increased groundwater evaporation capacity, caused soil salinization, and increased groundwater salinity [15].

The groundwater recharge quantity of Luoyang Basin is 3.3–4.1 × 10^8^ m^3^/a (from 1995 to 1999) and the exploitation quantity is 3.8–4.3 × 10^8^ m^3^/a (from 1996 to 2000). The substantial decline in groundwater levels and the deterioration in quality were due to annual local overexploitation. In order to meet needs for groundwater as a resource and beautify the urban environment, five rubber dams were built on the Luohe River from 2000 to 2008. This paper discusses the impact of the rubber dam construction on groundwater and provides references for groundwater environment management and protection of Luoyang Basin.

## 2. Regional Physical Geography and Hydrogeology

### 2.1. Regional Physical Geography

**Figure 1:**
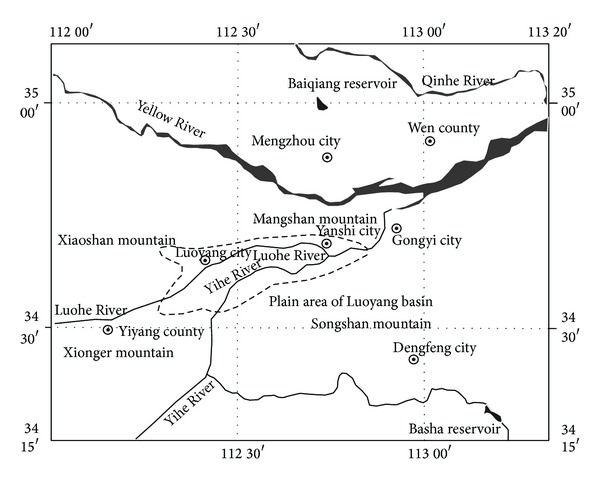
Luoyang Basin map.

The Luoyang Basin is located in western Henan Province, surrounded by Mang Shan, Xiao Shan, Xionger Shan, and Song Shan (Figure 1). It has a warm-temperate and monsoon climate. According to meteorological data, the perennial average temperature is 14.3°C and multiyear evaporation is 1451.7 mm. The multiyear average precipitation is 545.98 mm and this is subject to considerable temporal and spatial change. Precipitation is concentrated in July, August, and September, accounting for about 50% of annual precipitation.

### 2.2. Regional Hydrogeology

The Luoyang Basin formed in the late Mesozoic. It is a complete hydrogeological unit, surrounded by mountainous and loess hills. The Yi-luohe River alluvial plain is in the middle of it. Distribution and occurrence regulations of groundwater were dominated primarily by meteorology, hydrology, topography, formation lithology, and geological structure. Precipitation infiltration is the main supplement to groundwater.

The south side of the Basin is wildly composed, with carbonate rocks, and the north and west side are a loess hilly area with a steep slope and developed a deep clough. This kind of landform lends itself easily to runoff and discharge. The groundwater in this area can run very short.

**Figure 2:**
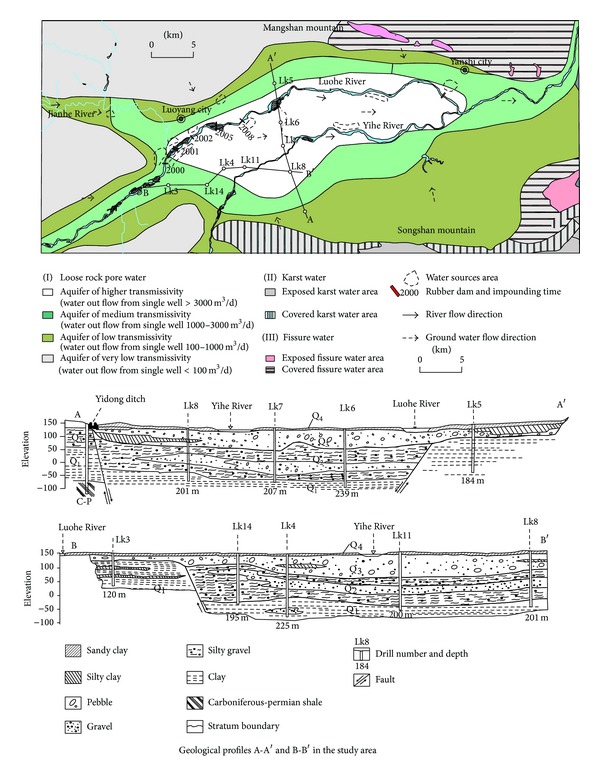
Hydrogeological plan fig. and profiles A–A′ and B–B′ in the Luoyang Basin.

The Yi-luohe River alluvial plain has subsided since the Quaternary period. It was the lowest part of this area and became a catchment area for surface water and groundwater. The aquifer of the whole Yi-luohe River alluvial plain area, especially in the massif and overbank, is thick. The aquifer is either directly exposed to the surface or covered by a minimal layer of earth or rock. This area has abundant groundwater resources. Generally, a single well can yield over 3000 m^3^/d. The first and second terraces are mostly presented with a dual structure, an upper layer covered with sandy clay, and a lower layer stock with coarse sand and sandy gravel. Here, a single well can yield 1000–3000 m^3^/d (Figure 2).

## 3. Primary Environmental Geological Problems before and after the Rubber Dam Construction

**Figure 3:**
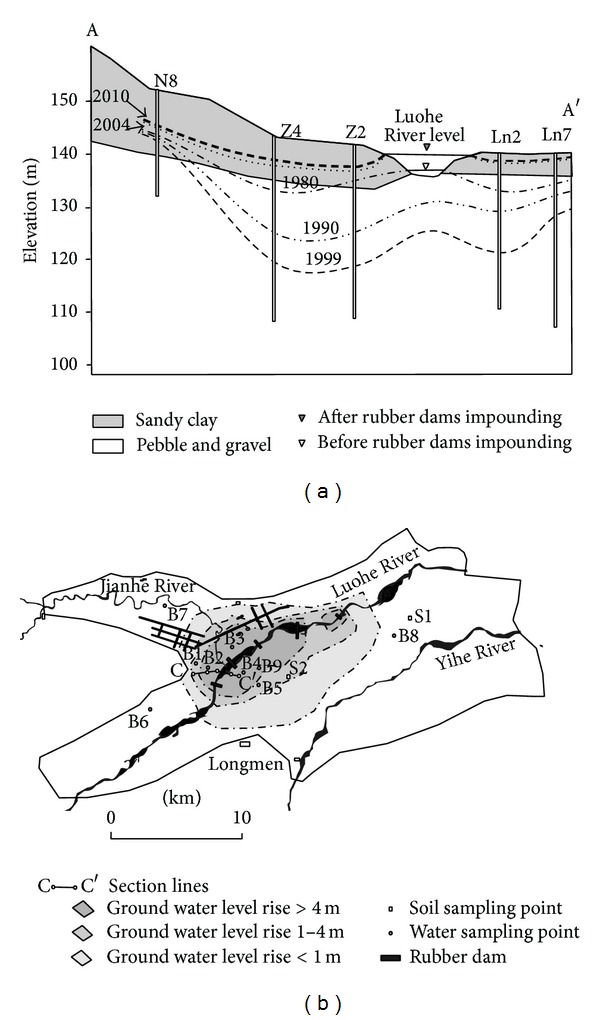
Changes of water tables before and after the rubber dam's construction.

From 1957 to present, there have been thirteen groundwater source fields found in the Luoyang Basin, the total yield reached 280–300 million m^3^/a. According to the monitoring data, the groundwater depth of the Luohe Riverside was 5.9–11.5 m in the middle and late 1980s. Because of overexploitation, the groundwater table has declined since the early 1990s. The Luohe River became a suspended river. Until 1999, groundwater depth was as low as to 13–19 m and even reached 30 m in some places (Figure 3(a)).

Due to long-term excessive exploitation of groundwater, the Luoyang Basin has suffered a serious environmental problem, including depression of the water table, deterioration of water quality, and land subsidence. In order to alleviate the increasingly intense groundwater shortages and improve the city landscape, the Luoyang city government built five rubber dams from 2000 to 2008 (Table 1 and Figure 3(b)).

Construction of Luohe River rubber dams has changed the regional groundwater flow system tremendously. The groundwater table along the river began to rise when the first rubber dam was built in April 2000. In addition, the cone of depression which had been caused by overpumping of groundwater along the river has already dispersed, resembling the state recorded in 2004. The main replenishment of the Luohe River to groundwater has changed from vertical seepage to lateral one (Figure 3(a)). As the groundwater level has risen, its quality has changed as well.

## 4. Materials and Methods

Nine observation wells were selected in Luoyang Basin, China, to study the variations in pH, total hardness (TH), total dissolved solids (TDS), nitrate nitrogen (NO_3_-N), and ammonium nitrogen (NH_4_-N) before and after the construction of the Luohe River rubber dams. The data were gathered over the course of 24 years from 1989 to 2012 and sampled in the dry period (October to December of each sampling year).The positions of the observation wells are shown in Figure 3(b) and the vertical dimension of the observation to the Luohe River is shown in Table 2. Observation wells number B6, number B7, and number B8 are located in an area largely unaffected by rubber dams. First, the water was left to run from sampling source for 4-5 min, before taking the final sample. Samples were collected in precleaned sterilized polyethylene bottles of 3 L capacity. The pH of the collected water samples was measured in the field using portable pH meter. TH was determined using ethylenediaminetetraacetic acid (EDTA) titration method. TDS were estimated by gravimetric method. NO_3_^−^ was determined in inductively coupled plasma mass spectrometry (ICP-MS). NH_4_^+^ was determined in ultraviolet-visible spectrophotometer.

To analyze characteristics of nitrogen distribution in the vadose zone, two undisturbed sediment cores were collected in October 2011. The cores were accomplished by drilling and sampling with a 7.5 cm diameter hollow stem hand auger. Two cores (core S1 and core S2) from two locations (Figure 3(b)) were taken continuously from the ground surface to a depth of 6.5 m. Bulk soil samples of w400 g were collected at different depths (Table 3). Samples were homogenized over the sampling interval and immediately sealed in polyethylene bags. Gravimetric moisture content was determined by drying a minimum of 100 g of soil sample at 110°C for 12 h. For the unsaturated zone moisture extracts, 200 g of sediment was combined with 200 mL of deionized water and shaken for 30 min at room temperature, after which a mixture of deionized water and soil water was produced using centrifuge at a rotational speed of 4000 rpm [16]. All of the samples were kept at 4°C prior to analysis.

## 5. Results

**Figure 4:**
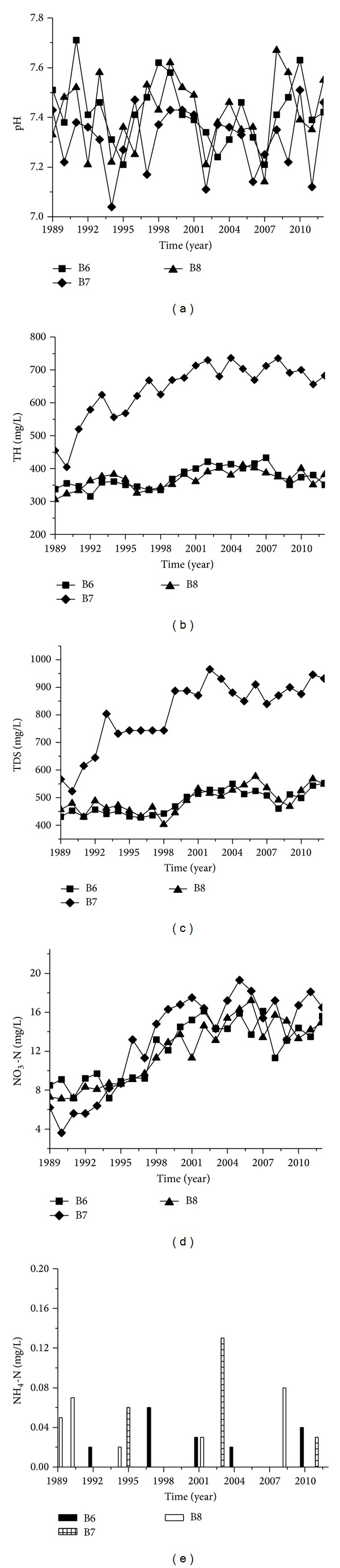
Concentration changes of pH, TH, TDS, NO_3_-N,and NH_4_-N of wells B6, B7, and B8.

The pH values and NH_4_-N concentrations for observation wells number B6, number B7, and number B8 did not show marked variations when compared with values prior to the construction of the Luohe River rubber dams (Figures 4(a) and 4(e)). However, TH (Figure 4(b)), TDS (Figure 4(c)), and NO_3_-N (Figure 4(d)) were slightly higher than before the dam had been constructed. The average concentrations of TH, TDS, and NO_3_-N were 34.7–120.1 mg/L, 71.5–178.3 mg/L, and 4.6–6.9 mg/L, respectively, after the rubber dams were built (Table 4). Number B7 was located in the Jianhe River area and all its indexes went up the fastest. Number B6 and number 8 were in the Luohe River area, which showed similar changes. In all, regional groundwater tended to deteriorate because of overexploitation and human activity.

**Figure 5:**
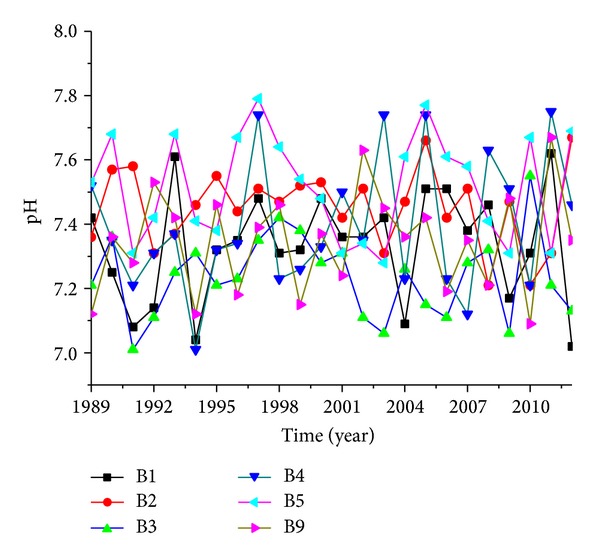
pH values change of groundwater of rubber dams affected area.

Observation wells number B1, number B2, number B3, number B4, number B5, and number B9 were located in the areas affected by rubber dams, and concentrations of TH, TDS, NO_3_-N, and NH_4_-N presented showed significant changes after the rubber dams were built but pH values did not change very much (Figure 5).

**Figure 6:**
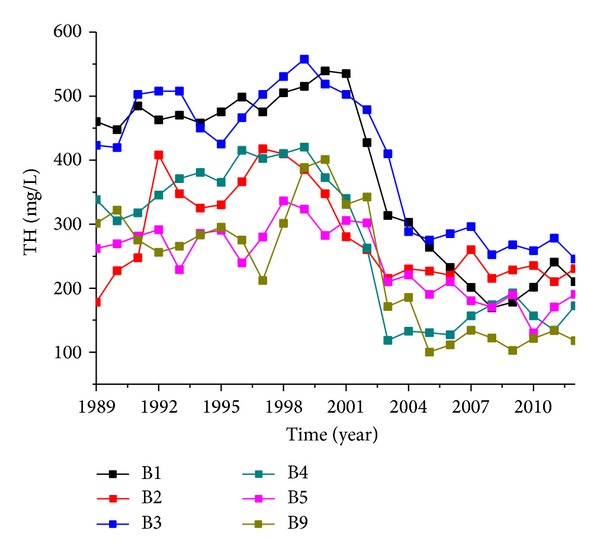
TH concentration changes of groundwater of the rubber dams affected area.

The annual average concentration of TH, TDS, and NO_3_-N declined within a range of 75.0–209.8 mg/L, 49.8–244.6 mg/L, and 0.2–14.0 mg/L, respectively, but the concentration of NH_4_-N increased within the range of 0.01–0.07 mg/L (Table 4). TH concentration of above wells obviously declined from 2000 and was then kept relatively stable (Figure 6).

**Figure 7:**
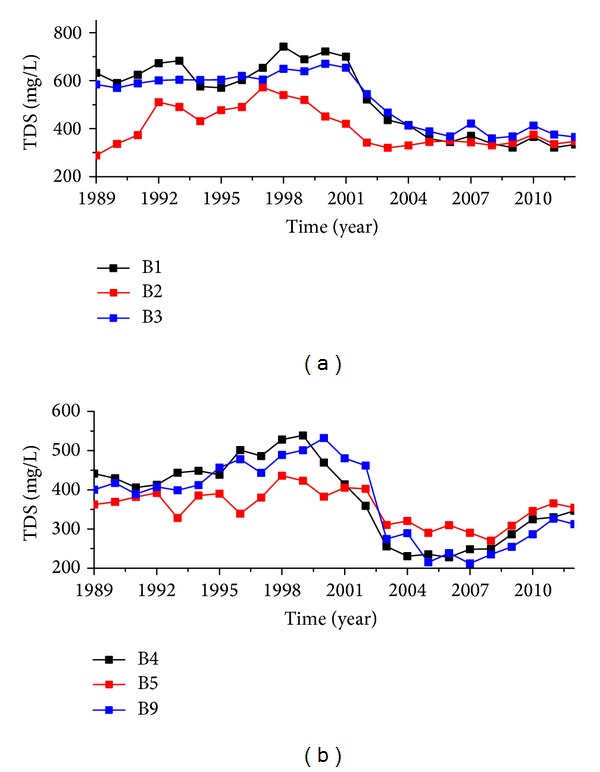
TDS concentration changes of groundwater of the rubber dams affected area.

Observation wells number B1, number B2, and number B3 are located on the first and second terraces of the north side of the Luohe River. The TDS concentration of these wells decreased from 2000, after which it became stable (Figure 7(a)). Number B4, number B5, and number B9 wells are located in the south bank of Luohe River floodplain. These rebounded after 2010 (Figure 7(b)).

**Figure 8:**
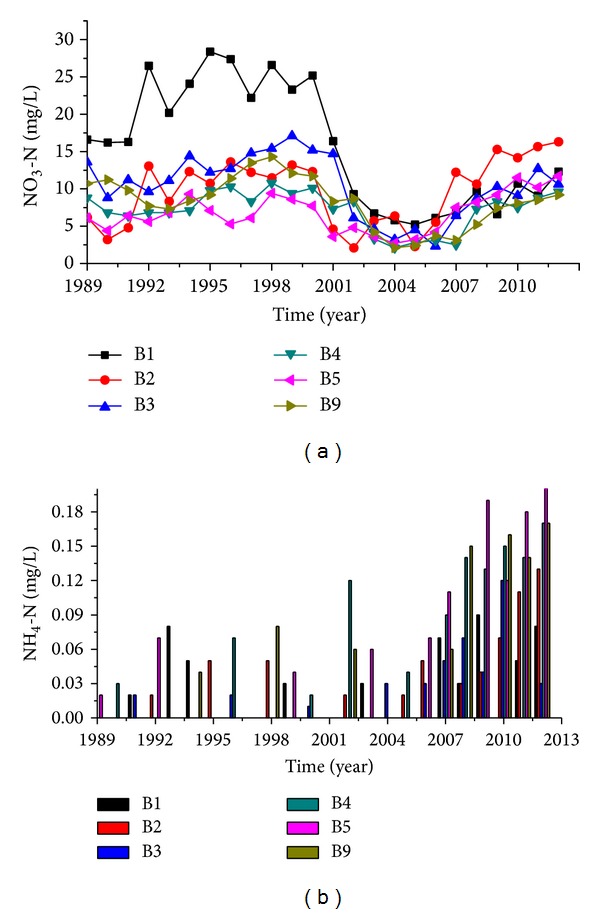
NO_3_-N and NH_4_-N concentration changes of groundwater of rubber dams affected area.

The concentration of NO_3_-N declined after 2000, following the construction of the rubber dam (Figure 8(a)). In most places, concentration was restored to its previous level since 2007. Number B2 well had higher levels during the study period than it had before the rubber dams were constructed.

The NH_3_-N concentration of the groundwater in this area was extremely low before the rubber dam was built. There have been twelve monitors from 1989 to 2000. Only number B1 was assessed four times and all other wells were assessed two or three times. The maximum concentration was 0.08 mg/L (Figure 8(b)). From 2000 to 2006, ammonia in the groundwater showed only small changes but it has increased since 2007, and the entire groundwater recovery has been mapped out (Figure 8(b)). The concentration peaked at 0.22 mg/L (number B5).

## 6. Discussions

### 6.1. Water Quality in Luohe River versus Regional Groundwater

The degree to which the water of the Luohe River has replenished the groundwater increased greatly after the construction of the rubber dams, so the water quality of the Luohe River has had a significant impact on that of the groundwater. According to the monitoring data of Luohe River water quality from 1998 to 2012, the values of TH, TDS, and NO_3_-N were lower than that of the regional groundwater, but the concentration of NH_4_-N and pH values were higher (Table 5). If the only action that takes place is mixing, then the concentrations of TH, NO_3_-N, and TDS in the groundwater would have exhibited similar changes and the concentration of NH_4_-N would be lower in groundwater than in the river. This may explain the concentration anomalies observed for NO_3_-N, TDS, and NH_4_-N in the groundwater following the rubber dam construction. This indicates that those changes not only had a close relationship with river replenishment but also had a close relationship with the local hydrogeological conditions and land use changes.

### 6.2. Concentrations of NO_3_-N and NH_4_-N in Groundwater

The concentration of nitrate in groundwater is affected by many factors, such as land use change groundwater replenishment, vadose zone lithology, nitrogen transformation in saturated zone and aeration zone, groundwater table depth, surface water and groundwater exchange, and groundwater dynamics [17–21].

The urbanization of Luohe River south bank has proceeded rapidly since 2005. A great deal of farmland has disappeared. Observation wells number B4, number B5, and number B9 are located in this area, which was farmland before 2007 and was replaced by buildings, parks, and wetlands as of 2010. This shows that the high levels of ammonia nitrogen and nitrate nitrogen in the groundwater could be more related to point sources of pollution than to agricultural activity.

According to soil sample analysis (Table 3), the difference in water-soluble NH_4_^+^ and NO_3_^−^ was enormous in the upper sandy clay and lower sandy gravel, which is in the vadose zone of the Luoyang Basin plain. Water-soluble NO_3_-N in the upper sandy clay was 2.43–18.25 mg/kg (dry soil), which was 0.11–0.36 mg/kg (dry soil) in the lower gravel. Water-soluble NH_4_-N in the upper sandy clay was found to be 19.6–41.6 mg/L (dry soil), and it was 2.3–4.1 mg/kg (dry soil) in the lower gravel.

Soil characteristics dictate nitrogen kinetics. In well-drained soils, infiltration is considerable, so the rate of nitrification is high and denitrification may be insignificant. Soil depth controls the time lag between the on-ground application of nitrogen and nitrate leaching and it influences the time span of soil nitrogen transformations [17].

The groundwater table rose from sandy gravel layer to the sandy clay layer after the rubber dams were built (Figure 3(a)). Water-soluble NO_3_^−^ and NH_4_^+^ in the vadose zone dissolved into the groundwater as the water table ascended, so the concentration of NO_3_^−^ and NH_4_^+^ increased gradually. The groundwater table is also subject to seasonal changes. Part of the sandy clay layer is in alternating drying-wetting situation that can accelerate nitrification. A part of NH_4_^+^ was converted to NO_3_^−^ in oxidizing atmospheres, causing a decrease in ammonia nitrogen in the groundwater, and the NO_3_^−^ concentration increased further. This process is affected by groundwater table depth, water level, and lithology of the water transition area. The nitrogen of Earth's surface reaches the groundwater in a shorter time due to superficial groundwater burial depth and speeds up the increasing rate of NO_3_^−^ [22].

The changes in NO_3_-N and NH_4_-N in this area are presented mainly in two phases: from 2000 to 2006, rivers replenished the groundwater in abundance, and the NO_3_-N and NH_4_-N of the groundwater were mainly influenced by the quality of river water, presenting a depressed tendency. As the groundwater table rose, the river water replenished to groundwater decreasingly. After 2007, the NH_4_^+^ and NO_3_^−^ of the aeration zone and Earth's surface were the primary causes of the increase in the concentration of NO_3_-N and NH_4_-N in groundwater.

### 6.3. Analysis of TDS Changes in the Groundwater

The concentration of TDS in the groundwater was controlled by the types and characteristics of the rock, the properties of the replenishing water, human activities, water-rock interactions, and evaporation. The south bank (location of observation wells number 1, number 2, and number 3) and north bank (location of observation wells number 4, number 5, and number 9) of the Luohe River had the same lithology distribution and were subject to similar human activities, so the main cause of the different concentrations of TDS may be attributed mainly to different groundwater burial depths. Groundwater evaporation capacity is affected by burial depth, lithology, and meteorological conditions, and evaporation generally increases as depth becomes shallower.

The plain area of Luoyang Basin is with a double structure, the upper layer is sandy clay, and the lower layer is sand and gravel. When the groundwater table rises to the sandy clay layer, which consists of small granules, capillary elevation and groundwater evaporation both increase.

A groundwater evaporation simulation was performed before and after a four-grade rubber dam system was constructed on Bai River of Nanyang City [23]. This area has hydrology and meteorology conditions similar to those of the Luohe River. After the four rubber dams were finished, the evaporation loss reached 5.02 million m^3^, about 3 times of the amount of evaporation prior to the construction of the dam. Observation wells number 1, number 2, and number 3 were located on the first and second terraces, and regional groundwater burial depth was 5–15 m. Observation wells number 4, number 5, and number 9 were on the flood plain of the Yi-luohe River. The groundwater burial depth there is 0.3–4 m. There was much more evaporation of the south bank than on the north bank of the Luohe River. The TDS concentration has definitely increased over the ten years since the rubber dams were built under strong evaporation conditions and water-rock interactions caused by wet-dry season changes. Surface pollutants discharged by human activities entered the aquifer in a shorter time than rubber dam constructed before due to the shallow groundwater burial depth. This might be another reason why the concentration of TDS increased.

## 7. Conclusions

The present study revealed that five rubber dams built in Luohe River from 2000 to 2008 have affected the groundwater environment a great deal. With the construction of the first rubber dam in 2000 and 4 additional dams built later, groundwater overdraft problem caused by groundwater exploitation was almost completely alleviated. The groundwater table ascended 30 m in some places, even as early as 2004. Concentrations of TH, TDS, and NO_3_-N decreased considerably after river water began to replenish the groundwater.

In 2007, seven years after the rubber dam was constructed, the amount of NH_4_^+^ and NO_3_^−^ that had been adsorbed in the aeration zone was instead turned into the aquifer. As a result, the concentrations of NH_4_-N and NO_3_-N in this area were elevated. TDS of the south side of the Luohe River increased under strong evaporation and water-rock interactions in the sandy clay layer starting in 2010. With the rise of the groundwater level, pollutants discharged by human activities reached the aquifer in a shorter time than before. This is another reason why the concentration of TDS, NH_4_-N, and NO_3_-N increased.

Developing means of exerting reasonable control over groundwater resource management to prevent groundwater overdraft should be the focus of groundwater research in this region.

## Figures and Tables

**Table 1:** Basic information regarding rubber dams in Luoyang.

Stage	Locations	Storage time	Water surface elevation (m)	Bottom elevation (m)	Maximum dam height (m)	Backwater length (m)	Water area ×10^4^ m^2^	Water storage ×10^4^ m^3^
First stage	Shangyang gong	April, 2000	140.5	136.5	4	3200	148	369
Second stage	Tongleyuan	April, 2001	135.6	132.1	3.5	2330	128	327
Third stage	Luoshenpu	April, 2002	131.6	127.6	4	3270	184	504
Forth stage	Zhoushan	April, 2005	130.3	126	4.3	2200	219	468
Fifth stage	Hualinyuan	April, 2008	127.7	123	4.5	3200	148	500

**Table 2:** Locations of observation wells along the Luohe River.

Well number	Vertical dimension to Luohe River (m)	Location	Well depth (m)
B1	1850	Second terrace	40
B2	960	First terrace	45
B3	700	First terrace	30
B4	800	Flood plain	27
B5	2330	Flood plain	29
B6	1820	Second terrace	33
B7	6410	Jianhe river watershed	76
B8	2730	Flood plain	35
B9	1160	Flood plain	21

**Table 3:** Analysis of the chemical composition of water-soluble NH_4_-N and NO_3_-N in different soil depth. Unit: mg/kg dry soil.

Sample	S1			S2		
Buried depth	NH_4_-N	NO_3_-N	Soil type	NH_4_-N	NO_3_-N	Soil type
0–10 cm	32.1	14.36	Sandy clay	28.1	18.25	Sandy clay
20–30 cm	27.2	7.53	Sandy clay	21.4	14.34	Sandy clay
50–60 cm	29.1	5.88	Sandy clay	20.1	7.21	Sandy clay
110–120 cm	41.6	2.43	Sandy clay	32.3	4.32	Sandy clay
240–250 cm	25.3	6.12	Sandy clay	28.1	8.44	Sandy clay
310–320 cm	36.7	4.32	Sandy clay	19.6	6.21	Sandy clay
450–460 cm	3.2	0.36	Sandy gravel	22.6	5.21	Sandy clay
550–560 cm	2.5	0.25	Sandy gravel	4.1	0.32	Sandy gravel
610–620 cm				2.3	0.18	Sandy gravel
650–660 cm				2.6	0.11	Sandy gravel

**Table 4:** Each index of annual average concentration before and after the rubber dams construction.

Well number		B1	B2	B3	B4	B5	B9	B6	B7	B8
pH	Average of 1989 to 2000	7.32	7.47	7.26	7.33	7.54	7.32	7.46	7.32	7.42
	Average of 2001 to 2012	7.35	7.43	7.21	7.46	7.49	7.37	7.38	7.30	7.41
	Difference	0.03	−0.04	−0.05	0.12	−0.05	0.05	−0.07	−0.02	−0.01

TH (mg/L)	Average of 1989 to 2000	482.9	332.6	484.5	370.5	280.9	298.1	349.8	580.6	348.9
	Average of 2001 to 2012	273.1	234.5	320.0	174.9	206.0	164.4	393.9	700.8	383.6
	Difference	−209.8	−98.2	−164.5	−195.6	−75.0	−133.7	44.1	120.1	34.7

TDS (mg/L)	Average of 1989 to 2000	646.6	456.8	611.6	462.0	380.9	443.8	447.6	719.5	456.1
	Average of 2001 to 2012	402.0	348.4	428.1	292.3	331.1	298.9	519.1	897.8	528.4
	Difference	−244.6	−108.4	−183.5	−169.7	−49.8	−145.0	71.5	178.3	72.3

NO_3_-N (mg/L)	Average of 1989 to 2000	22.8	10.1	13.0	8.5	6.9	10.6	9.8	9.7	9.3
	Average of 2001 to 2012	8.7	9.2	7.8	5.9	6.7	5.9	14.5	16.7	14.5
	Difference	−14.0	−0.9	−5.2	−2.5	−0.2	−4.7	4.6	6.9	5.2

NH_4_-N (mg/L)	Average of 1989 to 2000	0.02	0.01	0.00	0.01	0.01	0.01	0.01	0.01	0.01
	Average of 2001 to 2012	0.03	0.04	0.03	0.08	0.08	0.06	0.01	0.01	0.01
	Difference	0.01	0.03	0.03	0.07	0.07	0.05	0.00	0.01	0.00

**Table 5:** Analysis of surface water quality.

Time	pH	TH (mg/L)	TDS (mg/L)	NO_3_-N (mg/L)	NH_4_-N (mg/L)
Oct. 1998	7.65	207.4	265.3	2.25	0.14
Oct. 1999	7.43	189.3	212.7	1.75	0.07
Oct. 2000	7.51	211.3	237.6	2.56	0.11
Oct. 2002	7.52	194.3	256.1	1.87	0.05
Nov. 2003	7.81	250.7	344.6	3.07	0.27
Oct. 2004	7.58	215.2	310.5	3.14	0.19
Nov. 2005	7.62	245.2	320.1	2.98	0.23
Oct. 2007	7.42	237.1	312.1	4.12	0.21
Nov. 2008	7.35	225.2	330.4	2.73	0.16
Nov. 2010	7.36	232.6	310.7	2.58	0.15
Oct. 2011	7.41	253.2	338.9	2.32	0.17
Nov. 2012	7.63	211.5	289.4	3.15	0.22
